# Comparison of two automated PGMI-based software tools for breast positioning quality assessment in DBT screening

**DOI:** 10.1186/s41747-026-00825-9

**Published:** 2026-10-13

**Authors:** Gisella Gennaro, Stefano Trazzi, Valentina Bordon, Vania Galli

**Affiliations:** 1 Veneto Institute of Oncology (IOV), IRCCS, Via Gattamelata 64, 35128 Padua, Italy; 2 Azienda USL Bologna, Bologna, Italy; 3 ULSS 6 “Euganea”, Padua, Italy; 4 AUSL Modena, Modena, Italy

**Keywords:** Automation, Breast density, Digital breast tomosynthesis, Mammography, Quality control

## Abstract

**Objective:**

Automated “Perfect/Good/Moderate/Inadequate” (PGMI)-based tools have been developed to standardize breast positioning quality assessment, but their agreement remains uncertain. This study compared two automated software systems applied to the same digital breast tomosynthesis (DBT) screening cohort.

**Materials and methods:**

This retrospective study included 778 women (3,112 views). Two software tools (SW1 and SW2) independently generated PGMI classifications and quality control (QC) feature outputs. Agreement was assessed using exact agreement and quadratic weighted *κ*. PGMI distributions were compared overall and by view, breast density, and breast volume. Associations between imaging parameters and PGMI scores were evaluated using Spearman correlation and ordinal regression.

**Results:**

SW2 assigned more *Inadequate* ratings than SW1 (10.9% *vs*. 0.7%, *p* < 0.001), whereas SW1 assigned more *Perfect* (21.9% *vs*. 12.9%, *p* < 0.001). Overall agreement was limited (48.2%; *κ* = 0.044, 95% CI 0.006–0.085). Agreement was higher in mediolateral oblique than in craniocaudal views. Across SW1-derived density strata, SW2 assigned more *Inadequate* and fewer *Perfect* ratings in higher density strata, with smaller differences observed at higher breast volume. Higher volumetric breast density and breast thickness were associated with lower PGMI scores, whereas breast volume showed positive associations, with the effect more pronounced for SW2. QC patterns differed markedly: SW1 most frequently flagged inframammary fold and posterior positioning criteria, whereas SW2 identified pectoral muscle and parenchymal coverage issues.

**Conclusion:**

Two automated PGMI systems showed limited agreement and systematic differences in classification behavior. These findings indicate a lack of interchangeability and support the need for harmonized definitions and validation frameworks for automated image quality assessment.

**Key Points:**

***Question*** Standardized automated assessment of breast positioning quality in digital breast tomosynthesis remains lacking, limiting comparability between different PGMI-based software tools in screening.

***Findings*** Two automated PGMI systems showed limited agreement and systematic differences across views, breast density, breast volume, and acquisition-related parameters in DBT screening.

***Relevance statement*** Automated PGMI systems are not interchangeable and reflect different operational definitions of image quality. Harmonized standards and rigorous validation are required before implementation in clinical quality assurance workflows.

**Graphical Abstract:**

**Figure:**
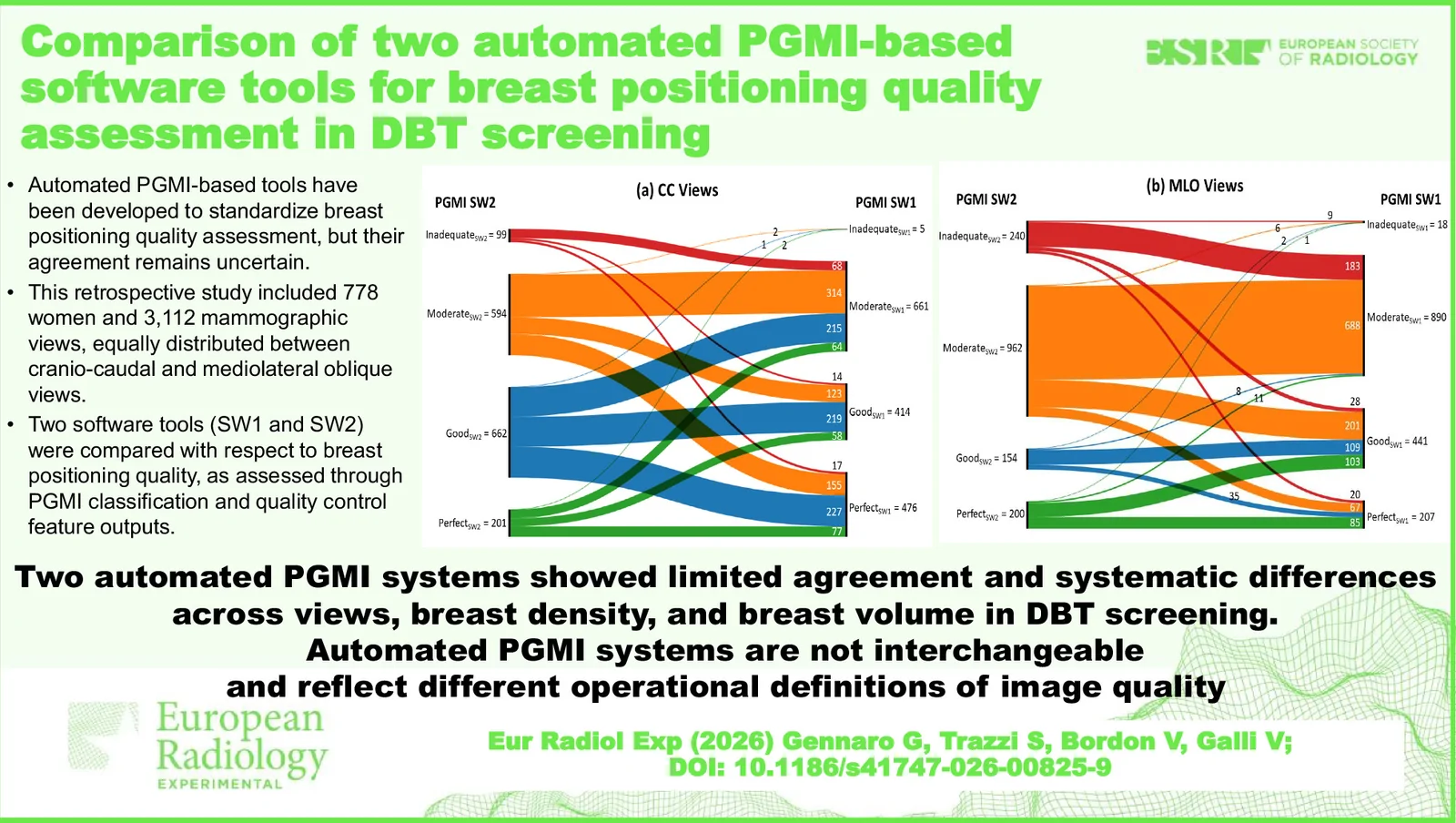

## Background

Adequate breast positioning is a fundamental prerequisite for high-quality mammographic imaging and directly affects lesion conspicuity, diagnostic accuracy, and radiation dose optimization. Suboptimal positioning may result in incomplete breast coverage, motion artifacts, skin folds and tissue superimposition, ultimately compromising cancer detection and recall rates [1–4]. Consequently, image quality assessment (IQA) remains a core component of quality assurance programs across all breast imaging modalities, including full-field digital mammography, digital breast tomosynthesis (DBT), and contrast-enhanced mammography, where positioning principles are largely shared. Robust IQA programs are essential for ensuring consistent image quality, optimizing diagnostic performance, and supporting accreditation within organized breast screening services [5].

Among the available frameworks for positioning-related IQA, the *Perfect*, *Good*, *Moderate*, *Inadequate* (PGMI) classification system is widely adopted in clinical practice and screening programs. PGMI provides a structured, ordinal assessment of positioning quality based on predefined anatomical and technical criteria and is commonly used for audits, training, and radiographer feedback [6–8]. However, PGMI assessment is inherently subjective, with well-documented inter- and intra-observer variability, even among experienced readers [9–13]. Differences in positioning assessment systems and local implementation of PGMI criteria further limit standardization across institutions, as demonstrated in international multicentre evaluations of mammographic positioning assessment [14]. This variability poses substantial challenges for standardization and limits the feasibility of large-scale quality monitoring based solely on human assessment.

Recent advances in artificial intelligence and image analysis have enabled the development of automated software tools for PGMI-like IQA. These systems aim to provide objective, reproducible, and scalable evaluations of positioning quality, potentially supporting continuous monitoring of image quality and radiographer training [7, 15, 16]. Typically, such approaches combine deep learning–based anatomical landmark detection (*e.g*., nipple position, pectoral muscle, inframammary fold) with rule-based or hybrid decision frameworks to derive a composite PGMI score. While these methods may reduce observer-dependent variability, they also introduce new challenges related to transparency and interpretability. Anatomical landmark detection algorithms, metric definitions, and decision rules are often proprietary and insufficiently documented, making it difficult to understand how individual quality features contribute to the final classification.

Despite increasing clinical availability, automated IQA tools differ substantially in algorithmic design, training data, and decision rules. Consequently, their outputs may not be directly comparable, and interchangeability cannot be assumed. In particular, differences in how systems operationalize positioning criteria, handle borderline cases, and respond to anatomical variability (*e.g*., breast size, density, and compression) may lead to systematic variations in PGMI classification, with implications for quality assurance and longitudinal monitoring.

Evidence from head-to-head evaluations of automated PGMI systems applied to the same dataset remains limited. Available data on inter-system concordance and on their sensitivity to acquisition and anatomical factors are scarce. A direct comparison is therefore needed to determine the level of agreement between systems and to characterize whether observed differences reflect distinct operational definitions of image quality rather than differences in underlying image content.

The PGMI-BENCH project was designed as a two-phase evaluation framework for automated PGMI-based IQA tools. The present study reports the first phase, which compares two commercially available software systems applied to a large DBT screening dataset. The analysis focuses on differences in PGMI classification, inter-software agreement, and associations with breast anatomy, acquisition parameters, and individual quality-control criteria contributing to the final score. The second phase will compare automated outputs with expert reader assessments.

## Methods

### Study design and ethical approval

This study (PGMI-BENCH) was conducted as a retrospective observational subanalysis of the RIBBS study, a personalized breast cancer screening program based on DBT, involving women aged 45–49 [17]. In the RIBBS screening program, women entered the screening pathway at approximately 45 years of age and were subsequently followed according to an individualized screening protocol until age 50. The present analysis was based on examinations from the first (prevalent) screening round; consequently, participants had only limited age variability around the target enrollment age. This recruitment design accounts for the narrow age distribution observed in the present cohort.

The RIBBS study was approved by the Ethics Committee of the Veneto Institute of Oncology (IOV) IRCCS, Padua, Italy, in May 2019 (approval code “RIBBS 2019/37), and written informed consent was obtained from all participants at enrollment. The present analysis was performed on pseudonymized imaging and metadata extracted from the approved study database, with direct patient identifiers removed and replaced by unique study identifiers, in accordance with institutional and national data protection regulations.

### Study population and dataset

The PGMI-BENCH study included all 785 DBT examinations recalled for further diagnostic assessment after double reading at the first screening round between 20 January 2020 and 21 December 2020. Examinations with incomplete outputs from either software system were excluded (*n* = 7), resulting in a final analytical cohort of 778 examinations (3,112 views), with equal representation of craniocaudal (CC) and mediolateral oblique (MLO) views.

### Automated software tools

Two automated software tools for breast positioning quality assessment were evaluated: *Volpara Lab v2.0.3* (Lunit International Ltd, Wellington, New Zealand) [18] and *b-box plus* (b-rayZ, Zurich, Switzerland) [19]. The two systems differ fundamentally in image input and processing pipeline. *Volpara* processes raw tomosynthesis projection images (DICOM “For processing”), whereas *b-box plus* analyzes processed synthetic 2D reconstructions generated from the DBT dataset (DICOM “For presentation”). *Volpara* is hereafter referred to as software 1 (SW1), and *b-box plus* as software 2 (SW2).

Both systems generate PGMI classifications based on predefined frameworks for breast positioning quality assessment, including evaluation of breast tissue coverage, nipple position, posterior and inferior breast inclusion, pectoral muscle visualization, inframammary fold depiction, and artifacts. Although conceptually aligned, implementation differs due to proprietary definitions, thresholds, and aggregation rules. Accordingly, individual QC features are considered system-specific operational implementations of shared conceptual domains.

A structured comparison of QC features across SW1 and SW2 is provided in Table 1.

**Table 1:** Quality control (QC) features and criteria used by the two software tools to generate the PGMI score

View	QC feature/criterion	SW1	SW2
CC	Nipple in profile	The nipple protrudes from the skin line	Nipple depicted/not depicted in profile
	Nipple position/orientation	The nipple is central and perpendicular in the breast, and is not exaggerated either medially or laterally ≥ 5°	Nipple axis outside ± 25° from the breast midline
	Posterior nipple line (PNL)	The CC PNL is no less than 1 cm shorter than the MLO PNL	Muscle depiction and absolute geometric threshold (20 mm) relative to MLO
	Breast tissue coverage	Medial or lateral breast tissue is not cut off	Complete/incomplete depiction of medial and lateral parenchyma
	Skin folds	Not evaluated in CC	Large/small/absent skin folds
MLO	Nipple in Profile	The nipple protrudes from the skin line	Nipple depicted/not depicted in profile
	Pec to PNL adequacy	The pectoral muscle extends inferiorly to at least 1 cm above the level of the PNL	The PNL level is met/not met
	Adequate pectoral/Pec shape	The vertical length is at least a 1/3 of the breast height, and the pectoral muscle angle is between 10° and 20°, and the pectoral shape is convex or straight	Pectoral muscle > 15°
	Breast tissue coverage	The breast tissue is not cut off inferiorly	Complete/incomplete depiction
	Skin folds	Linear skin folds on the pectoral muscle	Large/small/absent skin folds
	IMF visible	The IMF is in view and open	IMF depicted/not depicted

*CC* Craniocaudal, *IMF* Inframammary fold, *MLO* Mediolateral oblique, *Pec* Pectoral muscle, *PGMI* Perfect/good/moderate/inadequate, *PNL* Posterior nipple line, *QC* Quality control

### Breast density, volume, and stratification strategy

Both software systems provide automated estimates of breast density and breast volume. To ensure consistency across subgroup analyses, breast density categories and breast volume measurements derived from SW1 were used as the reference framework for stratification. SW1 estimates volumetric breast density using a physics-based model applied to raw mammographic data [20] and provides automated density categories based on these measurements. For the present analysis, these SW1-derived density categories (labeled a–d) were used for stratification and are referred to as SW1-derived density strata to distinguish them from radiologist-assigned BI-RADS breast composition categories. This approach provided a uniform basis for stratification across systems and avoided inconsistencies arising from differences in density and volume estimation algorithms.

### Image processing and analysis pipeline

All examinations were processed retrospectively using both software systems without manual intervention. Default configurations were applied in both tools, without recalibration or parameter modification. Each system independently generated PGMI classifications and QC feature outputs based on its proprietary algorithms and input image type.

Analyses were performed at the image level, the primary unit of PGMI-assessment in clinical practice. Although both software tools also provide examination-level scores obtained by aggregating image-level classifications, these aggregation procedures are proprietary and differ between systems. To ensure a like-for-like comparison of algorithmic performance, only individual mammographic views were included in the analysis.

### Statistical analysis

Statistical analyses were performed using R version 4.5.2 (R Foundation for Statistical Computing, Vienna, Austria) within RStudio (2026.04.0 Build 526; Posit Software, PBC, Boston, MA, USA). The analysis used the packages *readxl*, *dplyr*, *tidyr*, *irr*, *boot*, *stats*, *DescTools*, *MASS*, and *knitr*.

Agreement between software tools was evaluated at the image level using exact agreement, agreement within ± 1 PGMI category, and Cohen’s weighted *κ* (quadratic weights). Notably, agreement within ± 1 category reflects ordinal proximity and should not be interpreted as clinical equivalence. Ninety-five percent confidence intervals for *κ* were estimated using bootstrap resampling (1,000 iterations). Differences in the proportions of images assigned to each PGMI category between the two software tools were assessed using paired McNemar tests, performed separately for each PGMI category. These analyses were conducted overall and stratified by mammographic view (CC and MLO), breast density category (SW1-based), and breast volume quartiles (SW1-based). This approach preserved the paired structure of the image-level comparison data, as each corresponding mammographic view was evaluated by both software tools. As these comparisons were exploratory and descriptive, no adjustment for multiple testing was applied.

Associations between PGMI scores and quantitative imaging parameters were evaluated using Spearman's rank correlation coefficient. Multivariable associations were assessed using proportional odds ordinal logistic regression (*MASS* package, function *polr*), including all predictors simultaneously. Results were reported as odds ratios with 95% confidence intervals. Effect estimates are reported using clinically meaningful unit increments (5% for volumetric breast density, 100 cm³ for breast volume, 10mm for breast thickness, 10 N for compression force, and 10kPa for compression pressure), without affecting the model structure or statistical inference.

Finally, differences in the distribution of PGMI-related features between software systems were evaluated descriptively and stratified by mammographic view, given that feature definitions and evaluation criteria differ between CC and MLO views.

All statistical tests were two-sided, and a *p*-value < 0.05 was considered statistically significant.

## Results

### Study population

Table 2 summarizes the characteristics of the PGMI-BENCH study population.

**Table 2:** Characteristics of the PGMI-BENCH study population

Characteristic	Value
Age (mean ± standard deviation)	45.28 ± 0.25 years
Menopausal status	
Premenopausal	664 (85.3%)
Perimenopausal	99 (12.7%)
Postmenopausal	15 (1.9%)
SW1-derived breast density category	
b^a^	15 (1.9%)
b^b^	186 (23.9%)
b^c^	273 (35.1%)
b^d^	304 (39.1%)
Risk category	
Low	449 (57.7%)
Intermediate	322 (41.4%)
High	7 (0.9%)
Recall outcome	
Cancers	57 (7.3%)
B3 lesions	46 (5.9%)
Negatives	675 (86.8%)

Data are given as mean ± standard deviation or absolute frequency (percentage). The narrow age distribution reflects the RIBBS study design, in which women entered the screening program at approximately 45 years of age; the present cohort was derived from the first (prevalent) screening round

*B3 lesions* Lesions of uncertain malignant potential, *SW1* Software 1

a, b, c, and d are assigned to each individual view according to volumetric breast density (VBD): a, VBD ≤3.5%; b,VBD 3.5% and ≤7.5%; c, VBD 7.5% and ≤15.5%; d, VBD 15.5%. The patient/examination-level classification is subsequently derived by logically combining the classification labels assigned to the individual views

The final analytical cohort comprised 778 women (mean age 45.3 ± 0.3 years), predominantly premenopausal (85.3%). SW1-derived breast density categories c and d accounted for 74.2% of examinations. The distribution of diagnostic outcomes included 57 cancers (7.3%), 46 B3 lesions (5.9%), and 675 negative examinations (86.8%).

The analysis included all 3,112 mammographic views, with equal distribution between CC and MLO views.

### PGMI distribution and agreement

Table 3 summarizes PGMI category distributions assigned by the two software tools, overall and stratified by view type (CC and MLO).

**Table 3:** Distribution of PGMI ratings for the two software tools, overall and stratified by mammographic view

View	PGMI score	SW1 *n* (%)	SW2 *n* (%)	*p-*value	Exact Agreement	Agreement ±1	Weighted *κ* (95% CI)
Overall (*n* = 3,112)	Inadequate Moderate Good Perfect	23 (0.7%) 1,551 (49.8%) 855 (27.5%) 683 (21.9%)	339 (10.9%) 1,556 (50.0%) 816 (26.2%) 401 (12.9%)	**< 0.001** 0.904 0.233 **< 0.001**	48.2%	87.7%	0.044 (0.006–0.085)
CC (*n* = 1,556)	Inadequate Moderate Good Perfect	5 (0.3%) 661 (42.5%) 414 (26.6%) 476 (30.6%)	99 (6.4%) 594 (38.2%) 662 (42.5%) 201 (12.9%)	**< 0.001** **0.008** < **0.001** < **0.001**	39.2%	83.7%	0.050 (0.003–0.100)
MLO (*n* = 1,556)	Inadequate Moderate Good Perfect	18 (1.2%) 890 (57.2%) 441 (28.3%) 207 (13.3%)	240 (15.4%) 962 (61.8%) 154 (9.9%) 200 (12.9%)	**< 0.001** **0.001** < **0.001** 0.697	57.3%	91.7%	0.101 (0.040–0.160)

The table reports image-level counts and percentages for each PGMI category (*n* = 3112 views), paired McNemar test *p-*values for category-wise differences, and agreement metrics, including exact agreement, agreement within ± 1 category, and quadratic weighted *κ* with 95% confidence intervals

*CC* Craniocaudal, *CI* Confidence interval, *MLO* Mediolateral oblique, *PGMI* Perfect/good/moderate/inadequate, *SW1* Software 1, *SW2* Software 2

*p*-values in bold indicate statistically significant differences between the tested values

Overall, SW2 assigned substantially more images to the *Inadequate* category than SW1 (10.9% *vs*. 0.7%), whereas SW1 more frequently classified images as *Perfect* (21.9% *vs*. 12.9%) (Table 3). *The Moderate* and *Good* categories showed similar overall distributions across systems. Exact agreement was 48.2%, while agreement within ± 1 PGMI category was 87.7%. Weighted κ indicated poor agreement (*κ* = 0.044; 95% CI: 0.006–0.085).

**Fig. 1:**
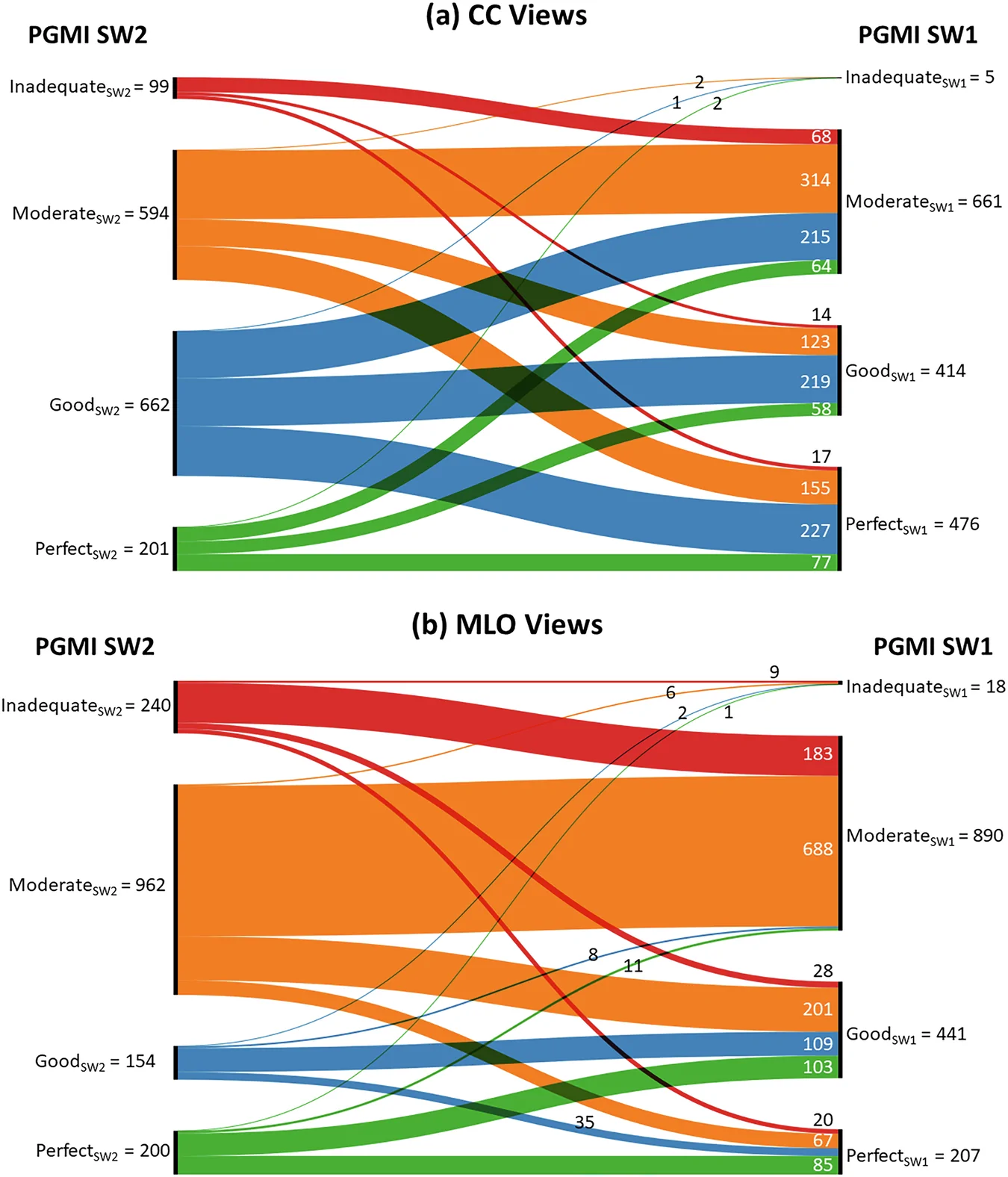
Sankey diagrams illustrating reclassification of PGMI scores between software tools for **a** craniocaudal (CC) and **b** mediolateral oblique (MLO) views (*n* = 3112 images). Flows are directed from SW2 (left) to SW1 (right). The width of each flow is proportional to the number of images transitioning between PGMI categories. PGMI, Perfect/good/moderate/inadequate; SW1, Software 1; SW2 Software 2

Agreement differed by view type, with lower exact agreement for CC than MLO views (39.2% *vs.* 57.3%), while agreement within ± 1 category remained high in both (83.7% and 91.7%, respectively). Across both views, disagreement followed a consistent pattern (Fig. 1): images classified as *Inadequate* by SW2 were predominantly reassigned as *Moderate* by SW1, whereas many images classified as *Perfect* by SW2 were downgraded to *Good* or *Moderate* by SW1. Disagreements spanning more than one PGMI category were uncommon.

**Fig. 2:**
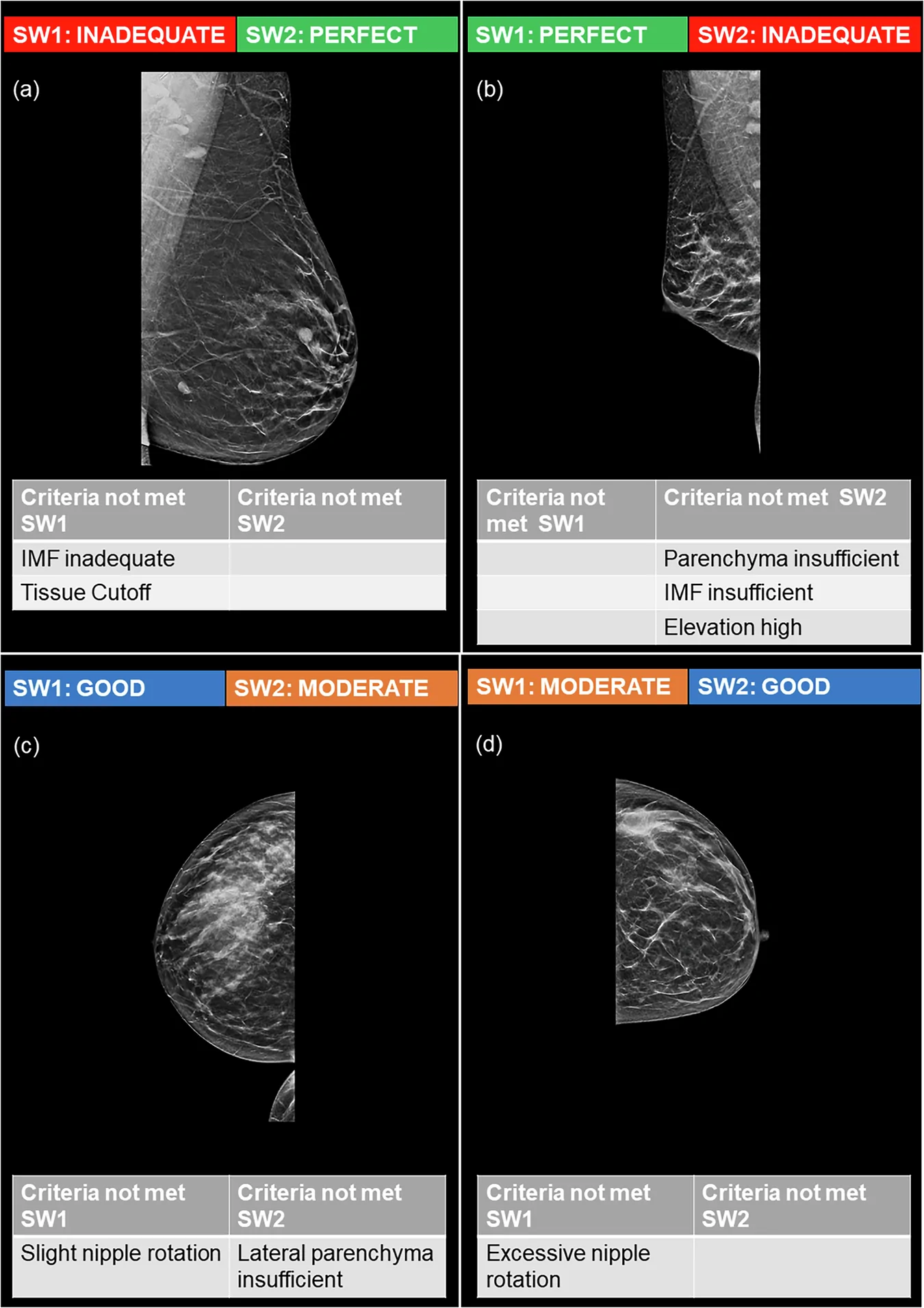
Representative examples of disagreement between SW1 and SW2, including the corresponding quality-control features flagged as not met in each case. **a** LMLO view classified as *Inadequate* by SW1 and *Perfect* by SW2; **b** RMLO view classified as *Perfect* by SW1 and *Inadequate* by SW2; **c** RCC view classified as *Good* by SW1 and *Moderate* by SW2; **d** LCC view classified as *Moderate* by SW1 and *Good* by SW2. The individual criteria flagged as not met are shown below the breast images for all scores other than *Perfect*. LCC, Left cranio-caudal; LMLO, left mediolateral-oblique; RCC, right craniocaudal; RMLO, right mediolateral-oblique; SW1, Software 1; SW2, Software 2

**Fig. 3:**
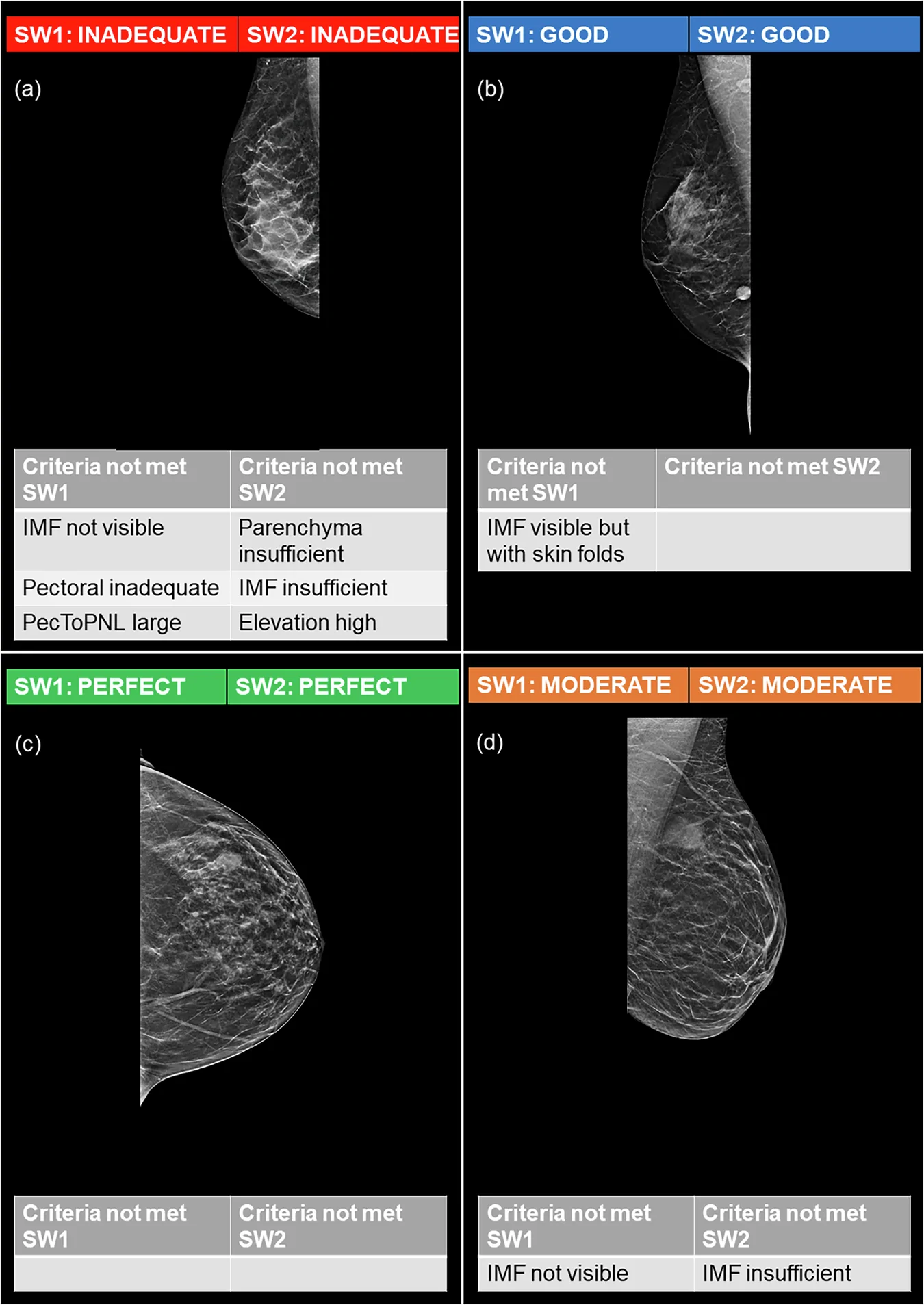
Representative examples of agreement between SW1 and SW2, including the corresponding quality-control features flagged as not met in each case. **a** RMLO view classified as *Inadequate* by both SW1 and SW2; **b** RMLO view classified as *Good* by both SW1 and SW2; **c** LCC view classified as *Perfect* by both SW1 and SW2; **d** LMLO view classified as *Moderate* by both SW1 and SW2. The individual criteria flagged as not met are shown below the breast images for all scores other than *Perfect*. LCC, Left cranio-caudal; LMLO, left mediolateral-oblique; RMLO, right mediolateral-oblique; SW1, Software 1; SW2, Software 2

Figures 2 and 3 illustrate representative cases of discordant and concordant classifications between systems.

### PGMI *vs.* breast density and volume

Table 4 reports PGMI distributions and agreement metrics between software tools across SW1-derived breast density and breast volume strata.

**Table 4:** Stratification of PGMI ratings by breast density and breast volume for the two software tools

Metric	Category	PGMI score	SW1 *n* (%)	SW2 *n* (%)	*p*-value	Exact Agreement	Agreement ± 1	Weighted *κ* (95% CI)
SW1-derived density strata	a (*n* = 134)	Inadequate	3 (2.2%)	1 (0.7%)	0.617	48.5%	88.8%	0.036 (-0.146–0.232)
		Moderate	52 (38.8%)	52 (38.8%)	1.000			
		Good	44 (32.8%)	45 (33.6%)	1.000			
		Perfect	35 (26.1%)	36 (26.9%)	1.000			
	b (*n* = 770)	Inadequate	5 (0.6%)	16 (2.1%)	**0.029**	50.5%	89.1%	0.081 (0.001–0.162)
		Moderate	328 (42.6%)	320 (41.6%)	0.646			
		Good	253 (32.9%)	263 (34.2%)	0.597			
		Perfect	184 (23.9%)	171 (22.2%)	0.417			
	c (*n *= 1,050)	Inadequate	8 (0.8%)	79 (7.5%)	**< 0.001**	49.7%	89.1%	0.015 (-0.054–0.082)
		Moderate	503 (47.9%)	536 (51.0%)	0.086			
		Good	294 (28.0%)	302 (28.8%)	0.714			
		Perfect	245 (23.4%)	133 (12.7%)	**< 0.001**			
	d (*n* = 1,158)	Inadequate	7 (0.6%)	243 (21.0%)	< **0.001**	45.3%	85.4%	0.046 (-0.023–0.108)
		Moderate	668 (57.7%)	648 (56.0%)	0.389			
		Good	264 (22.8%)	206 (17.8%)	**0.001**			
		Perfect	219 (18.9%)	61 (5.3%)	< **0.001**			
Breast volume (cm^3^)	Q1 [39.1, 385]	Inadequate	3 (0.4%)	163 (20.9%)	**< 0.001**	46.2%	84.3%	0.025 (-0.066–0.108)
		Moderate	476 (61.1%)	467 (59.9%)	0.662			
		Good	155 (19.9%)	110 (14.1%)	**0.001**			
		Perfect	145 (18.6%)	39 (5.0%)	< **0.001**			
	Q2 [385, 606]	Inadequate	5 (0.6%)	123 (15.8%)	< **0.001**	47.2%	87.9%	0.053 (-0.022–0.129)
		Moderate	425 (54.6%)	400 (51.3%)	0.164			
		Good	191 (24.5%)	209 (26.8%)	0.272			
		Perfect	158 (20.3%)	47 (6.0%)	**< 0.001**			
	Q3 [606, 939]	Inadequate	8 (1.0%)	37 (4.8%)	< **0.001**	48.6%	88.5%	0.030 (-0.045–0.105)
		Moderate	350 (45.1%)	368 (47.4%)	0.288			
		Good	241 (31.1%)	238 (30.7%)	0.906			
		Perfect	177 (22.8%)	133 (17.1%)	**0.003**			
	Q4 [939, 3,710]	Inadequate	7 (0.9%)	16 (2.1%)	0.095	50.9%	90.1%	0.064 (-0.022–0.143)
		Moderate	300 (38.6%)	321 (41.3%)	0.173			
		Good	268 (34.4%)	259 (33.3%)	0.642			
		Perfect	203 (26.1%)	182 (23.4%)	0.186			

Stratification was based on SW1-derived breast density strata (*a*–*d*) and SW1-derived breast volume quartiles. The table reports image-level counts and percentages for each PGMI category (*n* = 3112 views), paired McNemar test *p*-values for category-wise differences, and agreement metrics, including exact agreement, agreement within ± 1 category, and quadratic weighted *κ* with 95% confidence intervals

*CI* Confidence interval, *PGMI* Perfect/good/moderate/inadequate, *SW1* Software 1, *SW2* Software 2

*p*-values in bold indicate statistically significant differences between the tested values

Across SW1-derived density categories, the largest inter-software differences were observed in the highest-density group. In the lower strata (a and b), category distributions were broadly comparable, with only minor differences in the frequency of *Inadequate* assessments. Agreement metrics were stable across these strata (exact agreement approximately 48–51%; ± 1 agreement approximately 88–89%), while weighted *κ* remained low (0.015–0.081). In higher-density strata, divergence became more evident, driven mainly by an increased rate of *Inadequate* classifications in SW2 and a corresponding reduction in *Perfect* ratings. This pattern was further amplified in the highest-density stratum (class *d*), where SW2 assigned substantially more *Inadequate* ratings and fewer higher-quality ratings than SW1, although *Moderate* classifications remained similar between systems. Despite these distributional differences, overall agreement decreased only modestly with increasing density, and *κ*-values remained in the low range across all strata.

When stratified by breast volume quartiles, no monotonic trend in agreement was observed. The lower breast volume quartiles showed the greatest discrepancies, with SW2 more frequently assigning *Inadequate* ratings and assigning fewer *Perfect* assessments than SW1. In the intermediate quartile, differences between systems persisted but were attenuated, particularly for *Moderate* and *Good* categories. In the highest-volume quartile, distributions converged, with no statistically significant differences across PGMI categories and the highest agreement metrics observed (exact agreement approximately 51%; ± 1 agreement approximately 90%; *κ* = 0.064). Overall, inter-software variability was greater in the lower breast-volume quartiles, whereas agreement was highest in the largest quartile.

### Quantitative variables and PGMI scores

**Fig. 4:**
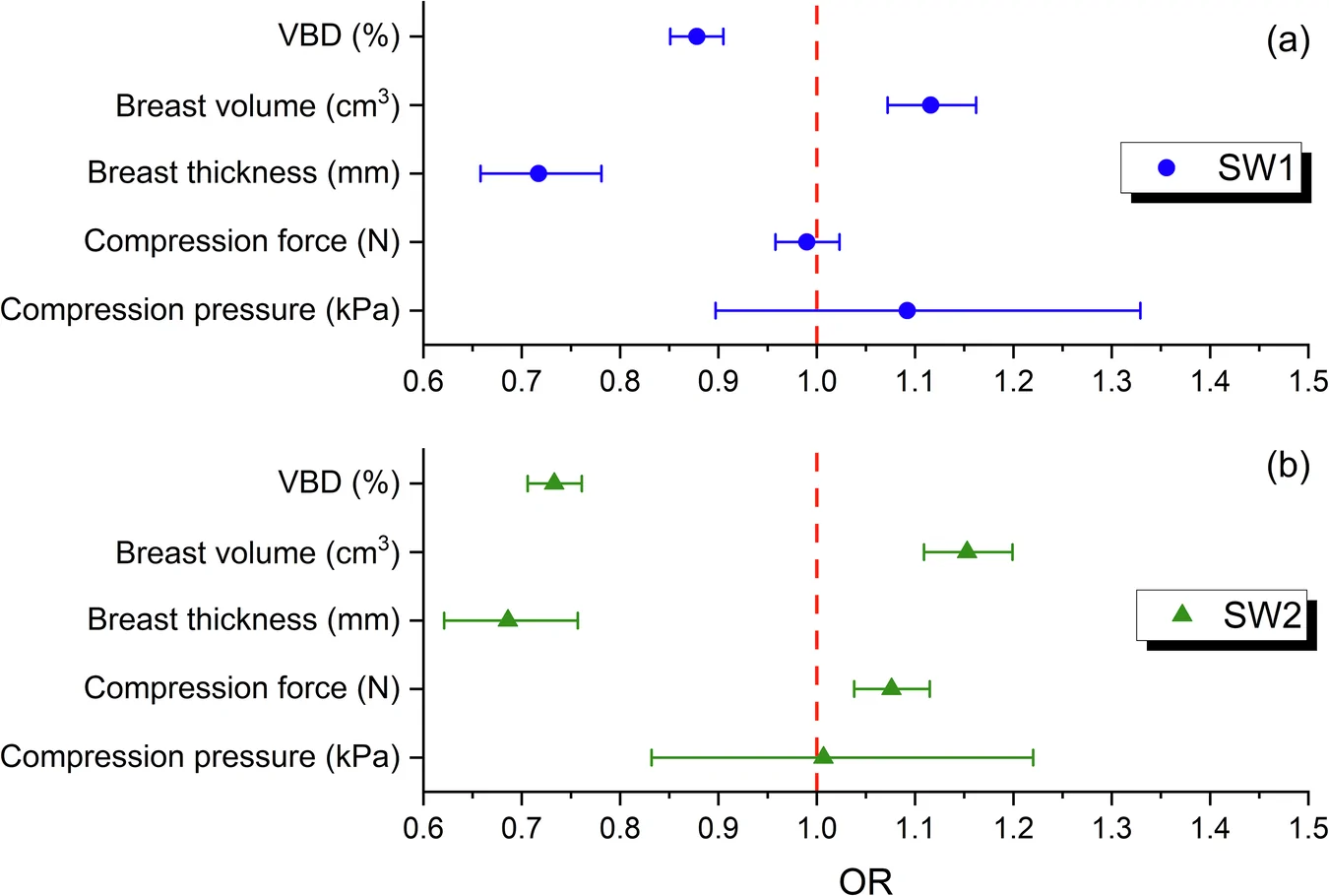
Forest plots of multivariable proportional odds ordinal logistic regression models for associations with PGMI scores in **a** SW1 and **b** SW2. Odds ratios (ORs) and 95% confidence intervals are reported for volumetric breast density (VBD%), breast volume, breast thickness, compression force, and compression pressure. ORs > 1 indicate higher odds of higher PGMI categories, whereas ORs < 1 indicate lower PGMI categories. The dashed vertical line indicates the null value (OR = 1). PGMI, Perfect/good/moderate/inadequate; SW1, Software 1; SW2, Software 2

Associations between quantitative imaging parameters and PGMI scores were consistent across analytical approaches, with generally stronger effects observed for SW2 than for SW1 (Table 5 and Fig. 4).

**Table 5:** Associations between quantitative imaging variables and PGMI scores

Variable	ρ_SW1_	ρ_SW2_	OR_SW1_ (95% CI)	p_SW1_	OR_SW2_ (95% CI)	p_SW2_
VBD (%) per + 5%	-0.121	-0.355	0.878 (0.851–0.905)	**< 0.001**	0.733 (0.706–0.761)	**< 0.001**
Breast volume (per + 100 cm^3^)	0.154	0.361	1.116 (1.072–1.162)	**< 0.001**	1.153 (1.109–1.199)	**< 0.001**
Breast thickness (per + 10 mm)	0.060	0.232	0.717 (0.658–0.781)	**< 0.001**	0.686 (0.621–0.757)	**< 0.001**
Compression force (per + 10 N)	0.063	0.231	0.990 (0.958–1.023)	0.545	1.076 (1.038–1.115)	**< 0.001**
Compression pressure (per + 10 kPa)	-0.064	-0.088	1.092 (0.897–1.329)	0.382	1.007 (0.832–1.220)	0.940

Univariate associations were assessed using Spearman's rank correlation coefficient. Multivariable associations were evaluated using proportional odds ordinal logistic regression models, with results reported as odds ratios and 95% confidence intervals. Effect estimates are expressed per clinically meaningful unit increments for each predictor, as specified in the “Methods” section

*CI* Confidence interval, *OR* Odds ratio, *VBD* Volumetric breast density

*p*-values in bold indicate statistically significant differences between the tested values

In univariate analysis, higher volumetric breast density was associated with lower PGMI scores in both systems, showing a weak correlation for SW1 (*ρ *= −0.121) and a moderate correlation for SW2 (*ρ* = −0.355). Breast volume showed positive associations with PGMI, again more pronounced for SW2 (*ρ* = 0.361) than SW1 (*ρ* = 0.154). Breast thickness and compression force were weakly positively correlated with PGMI in SW1 and more clearly in SW2, whereas compression pressure showed weak negative correlations in both systems.

Multivariable ordinal logistic regression confirmed these associations after adjustment for all covariates (Fig. 4). In both SW1 and SW2, increasing volumetric breast density and breast thickness were independently associated with lower odds of higher PGMI categories, while breast volume was associated with higher PGMI scores. These effects were more pronounced in SW2. In SW2 only, compression force showed an independent positive association with PGMI, whereas compression pressure was not significant in either model. Overall, multivariable modeling indicated a stronger dependence of SW2 outputs on breast composition and acquisition-related parameters compared with SW1. Odds ratios are reported using clinically meaningful unit increments (Table 5).

### Individual criteria flagged as not met

**Fig. 5:**
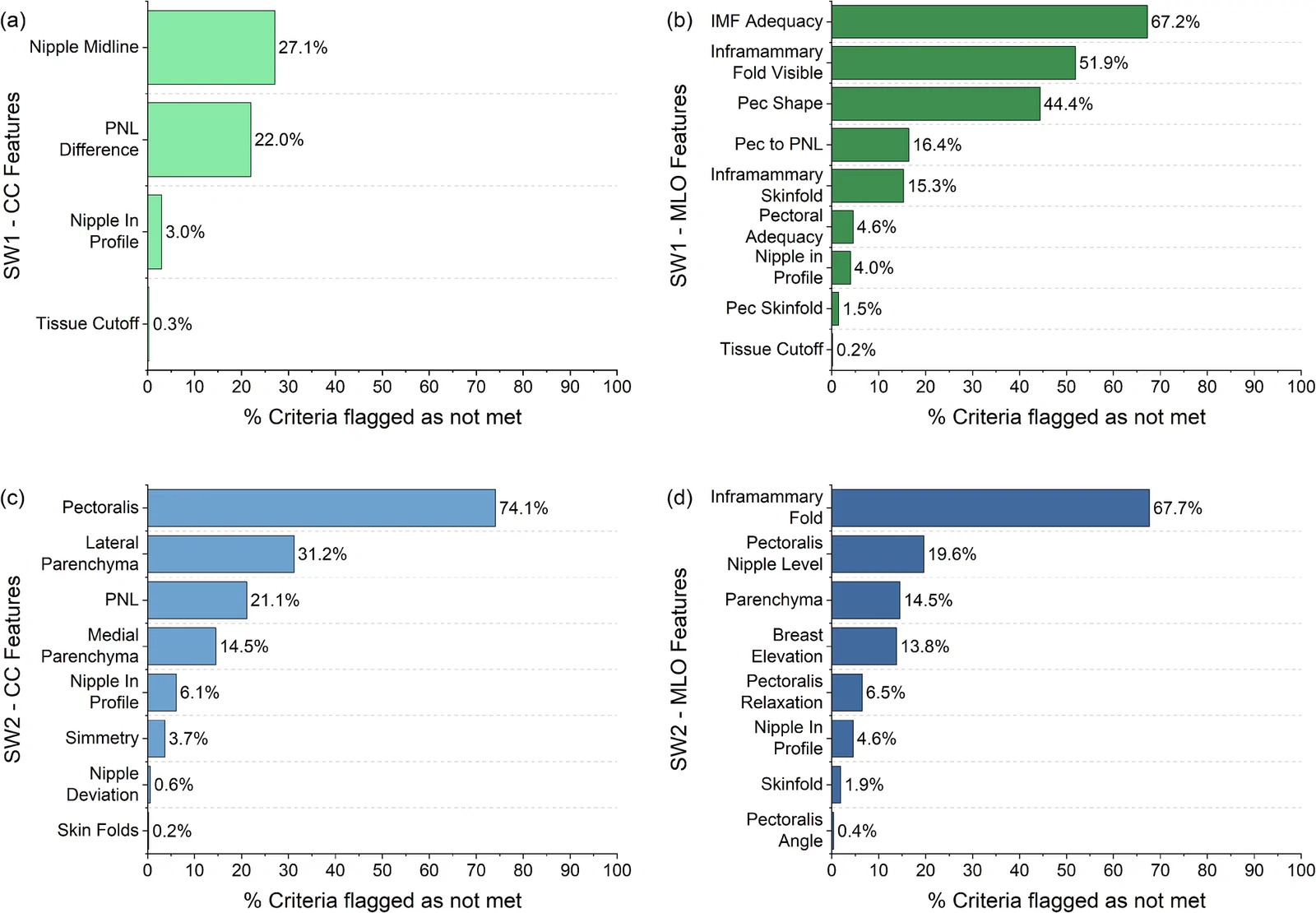
Distribution of quality control (QC) criteria flagged as not met by the two software tools. **a** SW1, craniocaudal (CC) views; **b** SW1, mediolateral oblique (MLO) views; **c** SW2, CC views; **d** SW2, MLO views. Values represent frequencies of flagged criteria. PNL, Posterior nipple line; SW1, Software 1; SW2, Software 2

Figure 5 summarizes the frequency of QC criteria flagged as not met by each software. In CC views, SW1 most frequently flagged nipple alignment and posterior coverage criteria, while SW2 predominantly flagged pectoral muscle depiction and lateral parenchymal coverage. In MLO views, both systems most frequently identified inframammary fold-related deficiencies; however, SW1 additionally emphasized pectoral geometry, whereas SW2 more frequently flagged pectoral nipple-level adequacy and parenchymal coverage. Overall, the two systems prioritized different anatomical features across CC and MLO views despite targeting similar positioning domains.

## Discussion

The PGMI-BENCH project was designed as a two-phase evaluation framework for automated PGMI-based IQA tools. The present study reports the first phase, comparing two commercially available software systems applied to a large DBT screening dataset. The analysis demonstrated limited agreement, systematic differences in category allocation, and distinct sensitivities to anatomical and acquisition-related parameters. Overall concordance was poor, with low weighted *κ*-values and moderate exact agreement, indicating that the two tools are not interchangeable despite their shared conceptual framework. Agreement remained consistently limited across craniocaudal and MLO views.

The combination of moderate exact agreement, high agreement within the ± 1 category, and low quadratic-weighted *κ* reflects marked differences in marginal PGMI distributions between systems. Under these conditions, agreement within adjacent categories remains relatively high due to clustering around boundary classifications, whereas weighted *κ* is more sensitive to systematic shifts in category allocation and prevalence effects. Therefore, the discrepancy between metrics should be interpreted as evidence of structured differences in grading behavior rather than random disagreement.

An additional source of discrepancy is the difference between image input and preprocessing pipelines. SW1 operates on raw tomosynthesis projection images, whereas SW2 analyses processed synthetic 2D reconstructions derived from the same DBT acquisition. Consequently, the systems do not evaluate identical image representations, and part of the observed variability likely reflects differences in reconstruction, contrast characteristics, and anatomical visibility inherent to the respective input domains. In this context, the comparison should be interpreted as an evaluation of two complete end-to-end IQA pipelines rather than a direct comparison of PGMI decision rules.

The observed discordance is consistent with the broader literature on PGMI assessment, which has repeatedly demonstrated substantial interobserver variability in manual scoring. Previous studies have reported low reliability for PGMI and related systems, with *κ*-values typically in the poor-to-fair range, even among trained readers [9, 10, 12, 13]. Variability has been observed both within and between institutions, reflecting differences in training, interpretation of anatomical landmarks, and local audit practices. The present findings extend this evidence by showing that variability persists even after removing human interpretation, indicating that inconsistencies are also embedded in algorithmic implementations of the same conceptual framework. Translating PGMI criteria into automated systems, therefore, remains challenging, particularly for anatomically ambiguous structures such as the pectoralis muscle and posterior nipple line [21].

The pattern of disagreement was structured rather than random, with systematic shifts between adjacent PGMI categories. This suggests that discrepancies arise primarily from differences in threshold definitions, feature weighting, and operational translation of anatomical criteria, rather than from fundamentally different detection of gross positioning errors. The consistent redistribution around boundary categories supports this interpretation [17].

Differences between views were limited. Although MLO views provide more consistent visualization of the pectoral muscle and inframammary fold and are generally considered more anatomically constrained than CC views [21], both views showed comparable patterns of disagreement, suggesting that view geometry alone does not explain the observed variability.

Positioning quality is inherently constrained by patient anatomy. The curvilinear thoracic geometry limits the complete projection of all breast tissue onto a planar detector, particularly at the medial and lateral margins [22]. Accordingly, several historical positioning benchmarks, including the “30% rule” for pectoral muscle visualization, were originally derived from expert consensus rather than empirical outcome-based validation. More broadly, many IQA frameworks in breast imaging remain expert-driven, reflecting the historical difficulty of large-scale annotation and outcome linkage [23]. Emerging evidence suggests that some established criteria may not consistently correlate with clinically meaningful endpoints, highlighting the need to reassess traditional standards [24]. Automated image analysis now enables more systematic evaluation of these features at scale [25, 26].

From a quality assurance perspective, poor positioning remains a major contributor to technical recalls and suboptimal examinations, with potential implications for downstream diagnostic workflow [27, 28]. Automated PGMI systems have been proposed for continuous quality monitoring, benchmarking, and feedback to radiographers [7, 29]. Reported implementations suggest potential improvements in technical performance and reductions in repeat imaging rates [17, 30]. However, the present results indicate that outputs from different systems are not directly comparable, limiting their interchangeability in longitudinal quality assurance frameworks and increasing the risk of artificial performance shifts following software changes. These findings support the need for harmonized definitions and external validation frameworks prior to routine clinical deployment.

Several limitations should be considered. The study was retrospective and based on a single screening cohort of relatively young women, which may limit generalizability. Both systems were evaluated as closed algorithms, preventing inspection of internal decision rules. The absence of a reference standard precludes assessment of absolute accuracy, and observed differences reflect relative disagreement rather than true performance. Analyses were performed at the image level to reflect the decision unit for positioning assessment, although this does not account for clustering within examinations. The cohort consisted of examinations recalled for diagnostic assessment within a large screening program; this provided a fully paired image-level dataset for algorithm comparison but may have introduced case enrichment and limited applicability to unselected screening populations. Breast density and breast volume parameters were derived from SW1 and subsequently used as common external covariates for stratified and regression analyses of both systems; although this approach was chosen for methodological consistency, it introduces a degree of asymmetry that should be considered when interpreting covariate-adjusted results. Finally, categorical agreement metrics do not fully capture the clinical implications of transitions between PGMI categories.

In conclusion, two automated PGMI-based quality assessment systems demonstrated limited agreement and systematically different behavior with respect to anatomical and acquisition-related factors. These findings indicate that current automated implementations of PGMI are not interchangeable and reflect differences in operational definitions rather than a unified measurement standard. Further harmonization and validation against expert reference standards are recommended before clinical integration into standardized mammographic quality assurance programs.

## Data Availability

The dataset analyzed during the current study is available in the Zenodo repository, http://zenodo.org/uploads/20701234#:~:text=10.5281/zenodo.20701234.

## Abbreviations

CC: Craniocaudal
DBT: Digital breast tomosynthesis
IQA: Image quality assessment
MLO: Mediolateral oblique
PGMI: Perfect/Good/Moderate/Inadequate
QC: Quality control
SW1: Software 1
SW2: Software 2
